# Tranexamic acid in high-risk shoulder arthroplasty patients: safety across thromboembolic, cardiac, renal, and neurologic risk profiles

**DOI:** 10.1007/s00402-026-06406-0

**Published:** 2026-07-03

**Authors:** Tarishi Parmar, Akin Adio, Asim Handy, Mohammad Daher, Alec Kellish, Adam Khan, Joseph Abboud

**Affiliations:** 1 Sidney Kimmel Medical College, Thomas Jefferson University, Philadelphia, USA; 2 Department of Shoulder and Elbow Surgery, Rothman Orthopaedics, Philadelphia, USA; 3 Perelman School of Medicine, University of Pennsylvania, Philadelphia, USA; 4 University of Pennsylvania, Philadelphia, USA; 5 Department of Shoulder and Elbow Surgery, Rothman Orthopaedics, Philadelphia, USA; 6 Department of Orthopaedic Surgery, Southern California Permanente Medical Group, Kaiser Permanente, Panorama City, USA

**Keywords:** Shoulder arthroplasty, Tranexamic acid, Blood loss, Transfusion, High-risk patients, Perioperative safety

## Abstract

**Introduction:**

Tranexamic acid (TXA) effectively reduces blood loss and transfusion requirements in shoulder arthroplasty. However, concerns regarding thromboembolic, neurologic, cardiac, and renal complications have limited its use in medically high-risk patients. This study evaluated temporal trends and the safety of TXA in high-risk patients undergoing total shoulder arthroplasty (TSA).

**Methods:**

A retrospective cohort study was conducted using TriNetX, identifying patients who underwent shoulder arthroplasty (2012–2025). Patients were stratified by preexisting high-risk conditions, including prior thromboembolism, renal failure, atrial fibrillation, seizure disorders, and visual disturbances. Within each subgroup, patients receiving TXA were propensity score–matched to those who did not. Perioperative TXA utilization trends were assessed. Ninety-day postoperative outcomes were compared, including transfusion requirements, thromboembolic events, cardiac complications, renal failure, neurologic events, infections, readmissions, emergency department visits, and mortality. Outcomes were reported as odds ratios (ORs) with 95% confidence intervals (CIs).

**Results:**

Reported perioperative TXA use began in 2012 and increased through 2025, with over 70% of patients in both standard- and high-risk cohorts receiving TXA. TXA use was associated with significantly lower transfusion rates (OR range 0.53–0.62) and readmissions (OR range 0.63–0.75) across all high-risk cohorts (all *p* ≤ 0.01). TXA use was not associated with increased risk of deep vein thrombosis, pulmonary embolism, stroke, or postoperative seizures in any subgroup. Notably, TXA demonstrated a protective association against myocardial infarction, cardiac ischemia, acute renal failure, and mortality in select high-risk populations. No increase in infection-related complications was observed.

**Conclusions:**

In patients undergoing shoulder arthroplasty, TXA use was safe across multiple high-risk medical populations and was consistently associated with lower transfusion and readmission rates, with reduced mortality in select cohorts. These findings support the broader use of TXA for blood conservation in shoulder arthroplasty, even among patients with traditionally high-risk comorbidities, while underscoring the need for future prospective, shoulder-specific safety studies.

**Level of evidence:**

III, Retrospective Cohort Study

**Supplementary Information:**

The online version contains supplementary material available at 10.1007/s00402-026-06406-0.

## Introduction

Shoulder arthroplasty is one of the fastest growing reconstructive procedures in the field orthopaedics and is associated with meaningful perioperative blood loss. Even a small hemorrhage in this setting can lead to severe postoperative anemia and result in a blood transfusion [8]. In contrast to its routine perception, transfusion is not benign. It has been independently linked to higher rates of infection, longer hospital stays, increased cost, and greater short-term mortality after shoulder arthroplasty [9]. As procedural volumes rise and the arthroplasty population becomes older and more medically complex, reducing blood loss has become an important and central goal of modern shoulder care [3]. 

Tranexamic acid has emerged as a reliable blood-sparing agent in shoulder arthroplasty and consistently reduces perioperative bleeding, hemoglobin decline, and transfusion requirements [3, 24]. Its growing use mirrors experience in hip and knee arthroplasty, where TXA has reshaped perioperative management and improved overall efficiency of care [16, 23]. In shoulder surgery, TXA offers a simple and effective means to limit blood loss and support early recovery, as well as reduce possible downstream complications associated with anemia and transfusion [3, 24]. For many surgeons, it has become a routine component of their protocols.

Despite this efficacy, there still remains concerns regarding TXA’s safety. There are many reported risks that include thromboembolic events, neurologic complications including seizures, cardiac morbidity and renal dysfunction [10]. These potential complications are especially concerning in patients with preexisting thromboembolic, cardiovascular, renal, or neurologic disease or patients with associated risks. As a result, many surgeons remain hesitant to administer TXA in these high-risk populations, even when the potential benefit of blood conservation is greatest [21]. 

While the literature clearly supports the efficacy of TXA in shoulder arthroplasty, data addressing its safety in medically high-risk patients remain limited. Most existing studies focus on relatively healthy cohorts and rarely stratify outcomes by comorbidity burden [1, 5, 24]. This leaves uncertainty in the very patients who may benefit most from TXA but also be the most vulnerable to adverse events. The present study evaluates the safety of TXA in shoulder arthroplasty across thromboembolic, cardiac, renal, and neurologic risk profiles using a large national database. By directly examining outcomes in these high-risk populations, this work seeks to clarify whether TXA can be used safely and consistently in patients most likely to benefit from its use. We hypothesized that TXA administration would not be associated with increased rates of thromboembolic, cardiac, renal, or neurologic complications in these high-risk patient populations.

## Methods

### Study database

This retrospective cohort study utilized the TriNetX Research Network (Cambridge, MA, USA), a large, federated database that aggregates de-identified electronic health records from numerous healthcare organizations across the United States. TriNetX captures diagnostic, procedural, and demographic data for 190 million unique patients and enables longitudinal outcome assessment through continuously updated encounter information. Because all patient data are anonymized in accordance with the Health Insurance Portability and Accountability Act (HIPAA), this analysis met criteria for exemption from institutional review board oversight.

### Patient selection

**Fig. 1:**
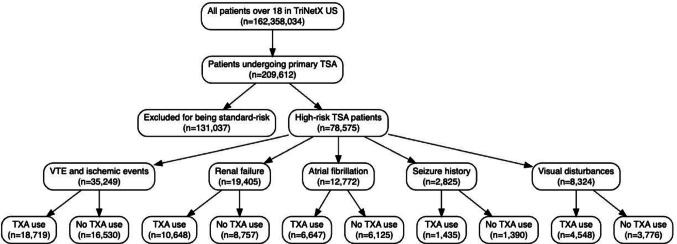
Study flow diagram illustrating cohort selection and high-risk subgroup stratification in total shoulder arthroplasty. *TSA* total shoulder arthroplasty, *VTE* venous thromboembolism, *TXA* tranexamic acid

A query was performed on January 15, 2025, using the TriNetX Research Network to identify adult patients who underwent primary total shoulder arthroplasty (TSA) between 2012 and 2025 using relevant Current Procedural Terminology (CPT) and International Classification of Diseases (ICD) codes. Tranexamic acid (TXA) exposure was identified using medication administration records. Patients were classified as high-risk for TXA use based on a documented history of at least one of the following conditions prior to TSA: thromboembolic disease (**ICD10-CM: I82**,** I26**,** I20-I25**,** I63**,** G45**), chronic kidney disease or renal failure (**ICD10-CM: N17-N19**), seizure disorder (**ICD10-CM: G40**), atrial fibrillation (**ICD10-CM: I48**), or visual disturbances (**ICD10-CM: H53**). Five separate high-risk cohorts were constructed corresponding to each condition (Fig. 1).

### 1:1 Propensity score matching

The TriNetX platform was used to perform 1:1 propensity score matching using logistic regression with nearest-neighbor matching and a caliper of 0.01. Balance between cohorts was assessed to ensure a standardized difference ≤ 0.01 for each covariate after matching. Propensity matching was conducted to account for demographic factors (age, sex, race) and relevant clinical comorbidities, including diabetes mellitus, tobacco use, rheumatoid arthritis, liver disease, overweight and obesity, alcohol-related disorders, opioid-related disorders, hypertensive diseases, heart failure, acute and chronic kidney disease, atrial fibrillation or flutter, epilepsy or recurrent seizures, long-term use of anticoagulant or antithrombotic/antiplatelet medications, and body mass index. Cohort pairs were also matched with respect to other high-risk classifications.

### Outcomes

The primary outcomes included 90-day postoperative complications following total shoulder arthroplasty. Medical outcomes assessed within 90 days included transfusion, deep vein thrombosis (DVT), pulmonary embolism (PE), myocardial infarction (MI), seizures, visual disturbances, ischemic stroke, cardiac ischemia, acute renal failure, emergency department (ED) visits, hospital readmission, and all-cause mortality. Surgical outcomes within 90 days included periprosthetic joint infection (PJI) and surgical site infection (SSI). All outcomes were identified using standardized International Classification of Diseases (ICD) and Current Procedural Terminology (CPT) codes available within the TriNetX platform.

First, temporal trends in TXA utilization were assessed by calculating the annual proportion of TSA patients receiving TXA among high-risk patients and among patients without high-risk conditions between 2012 and 2025, with no documented use of TXA prior to the study period. Second, within each high-risk subgroup, outcomes were compared between patients who received TXA and those who did not.

### Statistical analysis

Statistical analyses were performed using the TriNetX platform. Relative risks (RRs), 95% confidence intervals (CIs), and p-values were calculated for all outcomes. Categorical variables were compared using chi-squared tests, and continuous variables using Student’s t-tests. Temporal trends were plotted using R 4.03. Statistical significance was defined as *p* < 0.05.

## Results

**Fig. 2:**
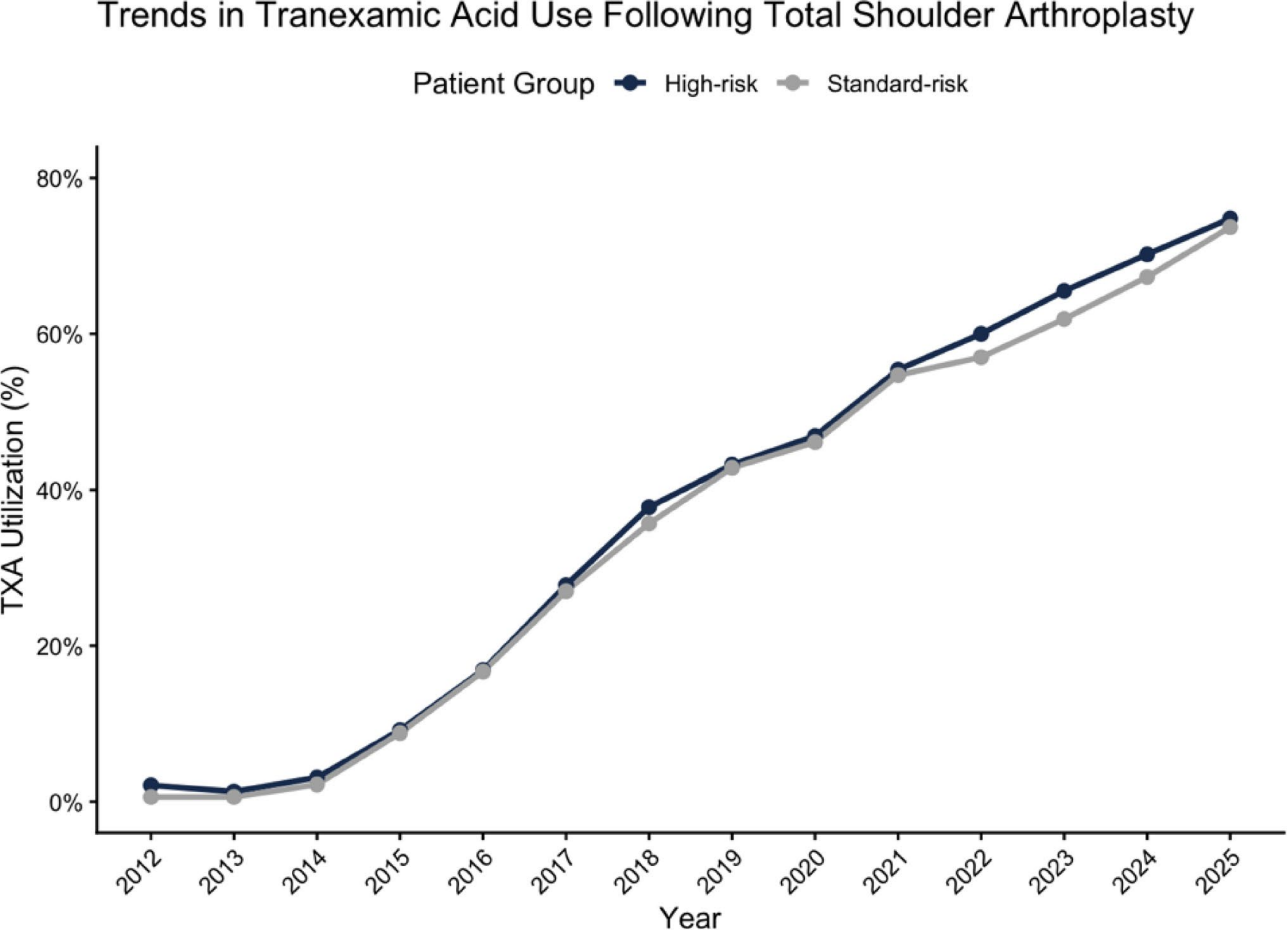
Temporal trends in tranexamic acid utilization following total shoulder arthroplasty among high-risk and standard-risk patients from 2012 to 2025

Between 2012 and 2025, utilization of tranexamic acid during primary total shoulder arthroplasty increased substantially over time among both high-risk and standard-risk patients (Fig. 2). In high-risk patients, TXA use rose from 2.1% of cases in 2012 to 74.8% in 2025, demonstrating a steady and pronounced upward trend throughout the study period. A similar increase was observed among standard-risk patients, with TXA utilization rising from 0.6% in 2012 to 73.7% in 2025.

### 90-Day outcomes in patients with prior thromboembolism

Following propensity score matching, body mass index differed between groups, with a mean BMI of 30.5 ± 6.5 in the TXA group and 31.0 ± 6.8 in the non-TXA group (*p* < 0.001). Among patients with a history of thromboembolism undergoing TSA (*n* = 16,702), tranexamic acid use was associated with significantly lower odds of several 90-day postoperative complications compared with no TXA use (Table 1). TXA use was associated with reduced odds of transfusion (OR 0.54; *p* < 0.001), deep vein thrombosis (OR 0.67; *p* < 0.001), pulmonary embolism (OR 0.68; *p* < 0.001), myocardial infarction (OR 0.67; *p* < 0.001), seizures (OR 0.73; *p* < 0.001), ischemic stroke (OR 0.84; *p* = 0.012), cardiac ischemia (OR 0.89; *p* < 0.001), acute renal failure (OR 0.73; *p* < 0.001), mortality (OR 0.48; *p* < 0.001), and hospital readmission (OR 0.63; *p* < 0.001). No significant differences were observed for visual disturbances, emergency department visits, periprosthetic joint infection, or surgical site infection (all *p* > 0.05).

**Table 1:** 90-Day outcomes in TSA patients with prior thromboembolism

90-Day outcome	TXA (%)	No TXA (%)	OR	95% CI	*P*-value
Transfusion	269	494	0.54	0.46–0.63	**< 0.001**
DVT	418	618	0.67	0.58–0.76	**< 0.001**
PE	239	351	0.68	0.57–0.81	**< 0.001**
MI	207	306	0.67	0.56–0.81	**< 0.001**
Seizures	247	336	0.73	0.62–0.87	**< 0.001**
Visual changes	147	134	1.1	0.86–1.41	0.45
Ischemic stroke	454	536	0.84	0.74–0.96	**0.012**
Cardiac ischemia	3272	3589	0.89	0.84–0.94	**< 0.001**
Acute renal failure	474	646	0.73	0.64–0.83	**< 0.001**
ED visits	1772	1,855	0.95	0.88–1.02	0.166
Mortality	70	149	0.48	0.35–0.64	**< 0.001**
PJI	264	276	0.96	0.80–1.14	0.615
SSI	144	124	1.17	0.91–1.51	0.219
Readmission	1233	1871	0.63	0.58–0.68	**< 0.001**

*DVT* deep vein thrombosis, *PE* pulmonary embolism, *MI* myocardial infraction, *TIA* transient ischemic attack, *PJI* peri-prosthetic joint infection, *ED* emergency department, *SSI* surgical site infection. Outcomes with significant difference betweeen TXA and no TXA cohorts (*P* < 0.05)

### 90-Day outcomes in patients with prior renal failure

Following propensity score matching, body mass index differed modestly between groups, with a mean BMI of 30.8 ± 6.8 in the TXA group and 31.4 ± 7.2 in the non-TXA group (*p* < 0.001). Among patients with a history of renal failure undergoing total shoulder arthroplasty (*n* = 8,845), tranexamic acid use was associated with significantly lower odds of several 90-day postoperative complications compared with no TXA use (Table 2). TXA use was associated with reduced odds of transfusion (OR 0.62; *p* < 0.001), pulmonary embolism (OR 0.59; *p* < 0.001), myocardial infarction (OR 0.75; *p* = 0.020), seizures (OR 0.71; *p* = 0.003), ischemic stroke (OR 0.81; *p* = 0.036), cardiac ischemia (OR 0.90; *p* = 0.010), acute renal failure (OR 0.77; *p* < 0.001), mortality (OR 0.63; *p* = 0.003), and hospital readmission (OR 0.64; *p* < 0.001). No significant differences were observed for deep vein thrombosis, visual disturbances, emergency department visits, periprosthetic joint infection, or surgical site infection (all *p* > 0.05).

**Table 2:** 90-Day outcomes in TSA patients with prior renal failure

90-Day outcome	TXA (%)	No TXA (%)	OR	95% CI	*P*-value
Transfusion	245	390	0.62	0.52–0.73	**< 0.001**
DVT	219	252	0.87	0.72–1.05	0.143
PE	96	161	0.59	0.46–0.78	**< 0.001**
MI	120	161	0.75	0.58–0.96	**0.02**
Seizures	147	205	0.71	0.57–0.89	**0.003**
Visual changes	84	83	1.01	0.74–1.39	0.935
Ischemic stroke	202	248	0.81	0.67–0.99	**0.036**
Cardiac ischemia	1344	1475	0.9	0.82–0.97	**0.01**
Acute renal failure	537	688	0.77	0.68–0.87	**< 0.001**
ED visits	1146	1151	1	0.91–1.09	0.925
Mortality	74	118	0.63	0.47–0.86	**0.003**
PJI	165	176	0.93	0.74–1.17	0.529
SSI	88	72	1.23	0.89–1.71	0.211
Readmission	865	1287	0.64	0.58–0.70	**< 0.001**

*DVT* deep vein thrombosis, *PE* pulmonary embolism, *MI* myocardial infraction, *TIA* transient ischemic attack, *PJI* peri-prosthetic joint infection, *ED* emergency department, *SSI* surgical site infection. Outcomes with significant difference betweeen TXA and no TXA cohorts (*P* < 0.05)

### 90-Day outcomes in patients with atrial fibrillation

Following propensity score matching, body mass index differed slightly between groups, with a mean BMI of 30.7 ± 6.6 in the TXA group and 31.0 ± 6.9 in the non-TXA group (*p* = 0.025). Among patients with a history of atrial fibrillation undergoing total shoulder arthroplasty (*n* = 6,184), tranexamic acid use was associated with significantly lower odds of several 90-day postoperative complications compared with no TXA use (Table 3). TXA use was associated with reduced odds of transfusion (OR 0.60; *p* < 0.001), acute renal failure (OR 0.82; *p* = 0.034), mortality (OR 0.59; *p* = 0.010), and hospital readmission (OR 0.75; *p* < 0.001). No significant differences were observed for deep vein thrombosis, pulmonary embolism, myocardial infarction, seizures, ischemic stroke or transient ischemic attack, cardiac ischemia, emergency department visits, periprosthetic joint infection, or surgical site infection (all *p* > 0.05).

**Table 3:** 90-Day outcomes in TSA patients with prior atrial fibrillation

90-Day outcome	TXA (%)	No TXA (%)	OR	95% CI	*P*-value
Transfusion	137	224	0.6	0.48–0.75	**< 0.001**
DVT	134	155	0.85	0.67–1.09	0.209
PE	71	98	0.72	0.52-1.00	0.05
MI	87	112	0.77	0.57–1.04	0.083
Seizures	79	98	0.8	0.59–1.10	0.175
Visual changes	60	59	1.02	0.70–1.49	0.923
Ischemic stroke / TIA	169	168	1.01	0.80–1.27	0.954
Cardiac ischemia	1,085	1,126	0.96	0.87–1.05	0.36
Acute renal failure	236	286	0.82	0.68–0.99	0.034
ED visits	708	709	1	0.89–1.12	0.953
Mortality	42	71	0.59	0.40–0.88	**0.01**
PJI	93	95	0.98	0.72–1.32	0.877
SSI	43	39	1.12	0.71–1.76	0.641
Readmission	596	772	0.75	0.66–0.84	**< 0.001**

*DVT* deep vein thrombosis, *PE* pulmonary embolism, *MI* myocardial infraction, *TIA* transient ischemic attack, *PJI* peri-prosthetic joint infection, *ED* emergency department, *SSI* surgical site infection. Outcomes with significant difference betweeen TXA and no TXA cohorts (*P* < 0.05)

### 90-Day outcomes in patients with prior seizure history

Following propensity score matching, baseline characteristics were well balanced between the TXA and non-TXA groups, with no statistically significant differences observed between groups (all *p* > 0.05). Among patients with a history of seizure disorder undergoing total shoulder arthroplasty (*n* = 1,456), tranexamic acid use was associated with lower odds of select 90-day postoperative complications compared with no TXA use (Table 4). TXA use was associated with reduced odds of transfusion (OR 0.53; *p* = 0.013), deep vein thrombosis (OR 0.54; *p* = 0.014), and hospital readmission (OR 0.68; *p* = 0.004). No significant differences were observed between groups for pulmonary embolism, myocardial infarction, recurrent seizures,

**Table 4:** 90-Day outcomes in TSA patients with prior seizure disorder

90-Day outcome	TXA (%)	No TXA (%)	OR	95% CI	*P*-value
Transfusion	28	53	0.53	0.32–0.88	**0.013**
DVT	30	54	0.54	0.32–0.89	**0.014**
PE	18	22	0.83	0.42–1.66	0.599
MI	18	23	0.79	0.40–1.56	0.49
Seizures	428	462	0.9	0.75–1.07	0.228
Visual changes	< 10	< 10	N/A	N/A	N/A
Ischemic stroke / TIA	58	63	0.92	0.61–1.38	0.68
Cardiac ischemia	158	173	0.9	0.70–1.17	0.436
Acute renal failure	49	67	0.73	0.48–1.11	0.141
ED visits	186	215	0.85	0.67–1.07	0.17
Mortality*	< 10	< 10	N/A	N/A	N/A
PJI	39	33	1.19	0.71-2.00	0.51
SSI	16	17	0.93	0.43–1.98	0.847
Readmission	132	186	0.68	0.52–0.88	**0.004**

*DVT* deep vein thrombosis, *PE* pulmonary embolism, *MI* myocardial infraction, *TIA* transient ischemic attack, *PJI* peri-prosthetic joint infection, *ED* emergency department, *SSI* surgical site infection

*Outcomes with < 10 reported patients were excluded from analysis per TriNetX reporting standards. Outcomes with significant difference betweeen TXA and no TXA cohorts (*P* < 0.05)

### 90-Day outcomes in patients with prior visual disturbances

Following propensity score matching, body mass index differed modestly between groups, with a mean BMI of 30.1 ± 6.6 in the TXA group and 30.6 ± 6.9 in the non-TXA group (*p* = 0.006). Among patients with a history of visual disturbances undergoing total shoulder arthroplasty (*n* = 3,806), tranexamic acid use was associated with lower odds of transfusion (OR 0.60; *p* = 0.004) and hospital readmission (OR 0.73; *p* < 0.001) compared with no TXA use (Table 5). No significant differences were observed between groups for deep vein thrombosis, pulmonary embolism, myocardial infarction, seizures, ischemic stroke or transient ischemic attack, cardiac ischemia, acute renal failure, emergency department visits, mortality, periprosthetic joint infection, or surgical site infection (all *p* > 0.05).

**Table 5:** 90-Day outcomes in TSA patients with prior visual disturbances

90-Day outcome	TXA (%)	No TXA (%)	OR	95% CI	*P*-value
Transfusion	56	92	0.6	0.43–0.85	**0.004**
DVT	94	95	0.99	0.73–1.33	0.939
PE	56	54	1.02	0.69–1.51	0.921
MI	44	59	0.74	0.491.12	0.15
Seizures	94	119	0.78	0.59–1.04	0.085
Visual changes	95	92	1.04	0.77–1.40	0.818
Ischemic stroke / TIA	117	147	0.79	0.61–1.02	0.07
Cardiac ischemia	481	508	0.94	0.82–1.08	0.379
Acute renal failure	99	124	0.8	0.60–1.05	0.106
ED visits	514	502	1.03	0.90–1.18	0.702
Mortality	11	19	0.55	0.26–1.20	0.13
PJI	49	56	0.88	0.59–1.32	0.542
SSI	37	26	1.46	0.87–2.46	0.15
Readmission	312	413	0.73	0.63–0.86	**< 0.001**

*DVT* deep vein thrombosis, *PE* pulmonary embolism, *MI* myocardial infraction, *TIA* transient ischemic attack, *PJI* peri-prosthetic joint infection, *ED* emergency department, *SSI* surgical site infection. Outcomes with significant difference betweeen TXA and no TXA cohorts (*P* < 0.05)

## Discussion

In this large database analysis of high-risk patients undergoing TSA administered TXA perioperatively, TXA was not associated with an increased risk of thromboembolic or major medical complications, even among patients with traditionally high-risk comorbidity profiles. Across all subgroups examined, TXA use was consistently associated with lower transfusion readmission rates and even lower mortality in select cohorts. These findings add to the growing body of evidence supporting the safety of TXA in shoulder arthroplasty populations beyond standard-risk patients.

### Trends in TXA administration over time

TXA utilization in shoulder arthroplasty increased steadily after 2012, with no recorded use in the TriNetX database prior to that year. Adoption rose similarly among standard- and high-risk patients, from 0.6% to 2.1% in 2012 to 73.7% and 74.8% by 2025, reflecting growing confidence in TXA’s safety and efficacy for blood conservation. Despite this increase, more than 30% of patients undergoing total shoulder arthroplasty did not receive TXA, mirroring trends in hip and knee arthroplasty where underutilization persists despite strong supporting evidence [21]. The continued underuse of TXA may reflect surgeon preference, institutional protocols, concerns regarding thromboembolic or neurologic risk, or reliance on alternative blood conservation strategies, including cell salvage, pharmacologic agents, surgical techniques, and perioperative fluid management [13, 21, 22]. Given the substantial evidence supporting TXA’s safety and efficacy, these findings highlight the need for broader adoption and standardized protocols in shoulder arthroplasty, while acknowledging the need for additional shoulder-specific safety studies [20–22]. 

### High-risk group 1: history of thrombosis

In patients with a prior history of thrombosis, TXA use was associated with significantly lower rates of transfusion, deep vein thrombosis, pulmonary embolism, myocardial infarction, mortality, and readmission. These findings are particularly noteworthy given the historical reluctance to administer TXA in patients with prior thromboembolic disease. Although TXA’s antifibrinolytic mechanism theoretically raises concern for thrombosis, contemporary evidence does not support an increased risk. Tauebeur et al.’s meta-analysis of 216 studies demonstrated no elevated thromboembolic risk across medical disciplines [25, 27]. Orthopedic studies similarly report no increased risk, with some noting protective effects against pulmonary embolism, myocardial infarction, and mortality [6, 7, 21]. In shoulder arthroplasty, Mayfield et al. likewise reported no increase in thromboembolic complications and reduced transfusion among 86,356 TSA patients with prior venous thromboembolism [18]. Collectively, these data indicate that a history of thrombosis alone should not preclude TXA use in shoulder arthroplasty, though individualized risk assessment remains essential.

### High-risk group 2: history of renal failure

Among patients with preexisting renal failure, TXA use was associated with lower rates of transfusion, acute renal failure, mortality, and readmission, without increased thromboembolic or neurologic complications. These findings are clinically relevant given the frequent inclusion of patients with kidney disease in orthopedic practice despite their unjustified exclusion from TXA trials [15]. Liu et al. similarly reported no increased thromboembolic or neurologic risk with peri-operative TXA use [15]. Isolated cases of TXA-related neurotoxicity in renal dysfunction, likely due to impaired clearance, have been described [17]. While no such events were observed in our cohort, these reports highlight the importance of appropriate dosing and the need for further study. The observed reduction in acute renal failure is supported by prior literature and may reflect indirect benefits of TXA, including reduced blood loss, improved hemodynamic stability, and decreased transfusion requirements [7]. 

### High-risk group 3: atrial fibrillation

In patients with atrial fibrillation, TXA use was associated with lower rates of transfusion, mortality, and readmission, without increased thromboembolic risk. These findings align with prior studies by Porto et al. and Tang et al. demonstrating no increased vaso-occlusive events and reduced transfusion in total joint arthroplasty (TJA) patients receiving TXA [21, 26]. Taken together, these data support the routine consideration of TXA in shoulder arthroplasty patients with atrial fibrillation, rather than its omission based solely on a history of arrhythmia.

### High-risk group 4: history of seizures

In patients with a prior seizure history, TXA use was associated with lower transfusion and readmission rates, without a significant increase in postoperative seizures or thromboembolic events. Although TXA can lower seizure thresholds via GABA-A and glycine receptor antagonism, particularly at high doses used in cardiac surgery, neurologic safety in orthopedic populations remains mixed [14]. Some TJA studies reporting no increased risk while others note higher seizure rates [11, 21]. To date, shoulder-specific studies have not demonstrated increased postoperative seizure risk with TXA [2]. Given this variability, higher-quality evidence is needed, consistent with the conclusions of the 2024 World Expert Meeting in Arthroplasty [7]. 

### High-risk group 5: history of visual disturbances

In patients with a history of visual disturbances, TXA use was associated with lower transfusion and readmission rates, without increased thromboembolic, visual, or neurologic complications. Although isolated reports describe transient visual disturbances, including blindness and color vision changes, after prolonged TXA use, these typically resolved after drug discontinuation [4, 12]. The exact pharmacodynamic mechanism underlying these effects remains unclear; however, adverse effects on retinal cone pigments have been proposed as a plausible explanation [4]. In contrast to our findings, Porto et al. reported increased risks of seizures and visual disturbances in total knee arthroplasty (TKA) patients with a prior history of visual disturbances who received TXA. This association, however, remains controversial and is markedly understudied in the current literature, warranting need for future research [7, 21]. 

Clinically, these findings support the consideration of TXA as a routine adjunct in total shoulder arthroplasty, even among patients with traditionally high-risk comorbidities. Careful patient selection and individualized risk assessment remain essential. Future research should prioritize prospective, shoulder-specific investigations to better define optimal dosing, routes of administration, and standardized perioperative protocols, as much of the current safety data continues to be extrapolated from hip and knee literature. Additional studies are required to clarify TXA’s neurologic safety profile in patients with seizure disorders or visual disturbances as well.

### Limitations

Several limitations should be considered when interpreting these findings. This study utilized the TriNetX federated electronic health record (EHR) network, which, while providing access to a large and diverse patient population, carries inherent limitations that are well recognized in the literature [19]. As a hospital-based EHR network, TriNetX captures a healthcare-seeking population and may not be fully representative of the general population. Patients may receive care across multiple institutions both within and outside the network, resulting in fragmented medical records. In addition, investigators cannot independently validate source records. Beyond these platform-specific considerations, this study shares limitations common to large administrative and EHR-based database research. Variability in diagnostic, procedural, and medication coding may have resulted in misclassification of TXA exposure, comorbidities, and postoperative outcomes. High-risk conditions represent heterogeneous clinical entities that could not be stratified according to severity, chronicity, disease control, or treatment status. Granular perioperative details were unavailable, including TXA dose, route, timing, frequency, and cumulative exposure. Similarly, information regarding surgical technique, implant type, operative time, estimated blood loss, anesthesia modality, perioperative fluid management, surgeon experience, and institutional blood-management protocols could not be assessed. Although propensity score matching was performed to balance measured baseline characteristics, residual confounding and confounding by indication remain possible because unmeasured factors influencing both TXA administration and postoperative outcomes cannot be accounted for. Outcomes were limited to complications identifiable through coded encounters within 90 days and did not include functional outcomes, patient-reported outcome measures, radiographic outcomes, or direct measures of blood loss. Finally, the retrospective observational design precludes causal inference. Therefore, the observed associations should be interpreted as supportive of the absence of an increased complication risk with TXA use in these high-risk populations rather than definitive evidence of safety or clinical benefit.

## Conclusion

In this large national database study of total shoulder arthroplasty, perioperative tranexamic acid use was not associated with increased thromboembolic, neurologic, renal, or major medical complications, even among patients with traditionally high-risk comorbidities. Across all high-risk subgroups examined, TXA use was consistently associated with lower transfusion and readmission rates, with reduced mortality observed in select cohorts. These findings support the safe use of TXA for blood conservation in shoulder arthroplasty acro098262

ss a broad spectrum of medical risk profiles and suggest that high-risk comorbidities alone should not preclude its use. Future prospective, shoulder-specific studies are warranted to further define optimal dosing strategies, administration routes, and neurologic safety in vulnerable populations.

## Supplementary Information

Below is the link to the electronic supplementary material.

Supplementary Material 1. Supplementary Table: ICD-10 and CPT codes used in the database

## Data Availability

The data that support the findings of this study were obtained from the TriNetX research network. Access to these data is subject to institutional licensing agreements and therefore the data are not publicly available. Data may be accessed by qualified researchers through TriNetX with appropriate institutional authorization.
