# Supplementary material for: Tranexamic acid in high-risk shoulder arthroplasty patients: safety across thromboembolic, cardiac, renal, and neurologic risk profiles
Source: Arch Orthop Trauma Surg. 2026 Jul 3;146(1):244. doi: 10.1007/s00402-026-06406-0 (PMC13331932; doi:10.1007/s00402-026-06406-0)
Supplement: Supplementary file 1 — Supplementary Material 1. Supplementary Table: ICD-10 and CPT codes used in the database [file 402_2026_6406_MOESM1_ESM.docx]

**Supplementary Table**

| **Codes** | |
| --- | --- |
|  |  |
| Total Shoulder Arthroplasty | 23472, 0RRJ0JZ, 0RRK0J7, 0RRK0JZ, 0RRJ00Z, 0RRK00Z |
|  |  |
| Tranexamic Acid | 10691 |
|  |  |
| **High Risk Cohorts** |  |
| Thromboembolic Disease | I82, I26, I20-I25, I63, G45 |
| Renal Failure | N17-N19 |
| Seizure Disorder | G40 |
| Atrial Fibrillation | I48 |
| Visual Disturbances | H53 |
|  |  |
| **Outcomes** |  |
| **90-day** |  |
| Transfusion | 302, 36430 |
| DVT | I82 |
| PE | I26 |
| MI | I21 |
| Seizures | G40, R56 |
| Visual Changes | H43 |
| Ischemic stroke/TIA | I63, G45 |
| Cardiac Ischemia | I20-I25 |
| Acute Renal Failure | N17, N19 |
| PJI | T84.5 |
| Readmission | Visit: Inpatient encounter |
| ED-visits | Visit: emergency |
| Mortality | Deceased |
| SSI | T81.4 |

DVT, Deep Vein Thrombosis; PE, Pulmonary Embolism; MI, Myocardial Infraction; TIA, Transient Ischemic Attack; PJI, Peri-prosthetic Joint Infection; ED, Emergency Department; SSI, Surgical Site Infection
